# Textural Flow Analysis of United States Commercially Available Baby Foods: Packaging and Delivery Method Comparisons by the International Dysphagia Diet Standardization Initiative Framework

**DOI:** 10.3390/foods14101771

**Published:** 2025-05-16

**Authors:** Larson P. Drzewicki, Donna R. Scarborough, Jeffrey D. Messinger, Michael Bailey-Van Kuren, Mickalyn S. Clemons, Memorie M. Gosa

**Affiliations:** 1 Eaton Regional Educational Service Agency, Charlotte, MI 48813, USA; larson.a.pax@gmail.com; 2 Department of Speech Pathology and Audiology, Miami University, Oxford, OH 45056, USA; scarbod@miamioh.edu; 3 College of Nursing, Fuld Institute for EBP Statistician, The Ohio State University, Columbus, OH 43210, USA; messinger.16@osu.edu; 4 Department of Emerging Technology in Business and Design, Miami University, Oxford, OH 45056, USA; 5 Connect Group LLC, Birmingham, AL 35216, USA; mickalynclemons@gmail.com; 6 Department of Communicative Disorders, University of Alabama, Tuscaloosa, AL 35401, USA; memorie.gosa@ua.edu

**Keywords:** commercial baby food labeling, feeding stages, food consistency, pediatric feeding

## Abstract

This study evaluated the flow and textural characteristics of commercial baby food in order to increase clinical knowledge to support patients with pediatric dysphagia. Samples from three organic and non-organic brands included four labeled stages and a variety of ingredients. A standardized method for evaluating the characteristics of room-temperature baby foods was utilized in order to compare, across two geographic regions, the brands and the labeled stages. Based on the manufacturing stages, no logical progression in thickness or texture was observed in relation to labeled food stages. Regardless of the stage, our results reveal that 75% of the baby foods samples are categorized as moderately thick liquid or liquidized food. Furthermore, two-thirds of products categorized as “large variability” foods were labeled as Stage 1. Caregivers and clinicians bear the burden for the presentation of safe and appropriate transitional foods during a child’s milk-weaning process. Current “staged” guidelines on baby foods do not accurately convey information about the product’s textural characteristics (i.e., thickness, cohesiveness, adhesiveness, etc.), which can influence the safety and efficiency of oral intake.

## 1. Introduction

During the first year of life, infants transition from a completely liquid diet of breastmilk or formula to a texture-rich diet that includes drinks and foods from the represented culture [1,2]. Infants in the United States are recommended to receive primary nutrition from breast milk or infant formula for the first 4–6 months of life [3], at which time more advanced textures can be introduced, called transitional feeding [4]. Transitional feeding in a healthy infant coincides with and is influenced by the interplay of neurophysiologic and anatomic maturation, changing nutritional demands, digestive enzyme development, as well as environmental, social, and cultural factors [5,6,7,8,9,10]. The successful navigation of this complex set of variables is necessary for the infant to learn to eat a variety-rich diet that will support rapid growth and development during early childhood [11]. Commercially available baby food provides one option for infants during this transition from liquids to more complex textures. The American Academy of Pediatrics (AAP) recommends an iron-fortified, single-grain infant cereal as the first food to be introduced via spoon when an infant is physically ready to begin complementary feeding. The AAP advises that cereal should be mixed with breastmilk or formula to a semi-liquid, runny consistency and fed from a small spoon. As the infant learns to extract the semi-liquid consistency from the spoon with increasing skill and efficiency, the AAP further advises increasing the texture: from smooth, runny pureed consistencies to thicker, creamier foods to food substances that contain pieces/chunks of solid foods. The AAP specifically points caregivers to commercial baby foods with increasing texture complexity as indicated by the manufacturer’s “labeled stages” (LSs) to determine the baby food texture that is right at each “developmental stage” (DS) [12]. The food texture represented by the LSs should be consistent, meaning Stage 1 foods should be predictably smooth and runny within all foods labeled as Stage 1 and across brands. Consistent LS labeling facilitates the presentation of food textures that can be safely and efficiently managed by the infant’s current oral–motor skills. Therefore, commercially available baby food labels should accurately communicate the texture of the food being offered and the corresponding oral feeding skill required for safe consumption.

Baby foods are also commonly used in treatment for infants that present with oropharyngeal dysphagia. Approximately 1% of infants and children in the general population will experience dysphagia [13], and the incidence of pediatric dysphagia is much higher (>80%) in some clinical populations, such as those with a history of prematurity [14]. Texture modification is a commonly recommended compensatory strategy for the management of pediatric dysphagia [15,16,17]. As infants and children recover from pediatric dysphagia, the use of modified foods and liquids must be gradually weaned to facilitate the eventual adoption of an age-appropriate diet, including regular foods [18,19,20]. The various textures of foods can be conceptualized in a hierarchy of increasing difficulty, moving from pureed foods that require little oral effort at the bottom to solid foods that require chewing at the top. Understanding the hierarchical progression of foods can assist in designing effective treatment plans that provide a gradual increase in the demand of oral–motor skills required to eat successfully. This hierarchical conceptualization of food textures not only promotes feeding skill progression in healthy, typically developing infants and children [1], but it also facilitates the safe transition to a regular diet after the resolution of dysphagia in pediatric populations. In commercially available baby foods, this hierarchical concept is manifested through the LSs, guiding caregivers in the selection of baby food [2,12]. The LS should help identify the right foods for their child’s DS (see Table 1). The LS might also hint at the ingredients within the package (i.e., Stage 2 foods might contain combinations of foods and not just single-ingredient foods, but carrying a Stage 2 label is not always indicative of combination purees). At this time, no governing agency or policy is in place to guarantee the LS corresponds to objective criteria that accurately describe the baby food texture or the ingredients included with that label. Additionally, the subjective descriptions of the texture of the baby foods are not uniform across cultures or across brands. However, it is important to consider both rheological features of the food, such as viscosity, flow rate, adhesion, and cohesion, as well as the potential impact on deglutition. Understanding the more complex interaction(s) of these rheological features across baby foods will allow clinicians to better serve these populations. Rheological testing can directly provide measures of some of these properties [21]. However, a rheometer is not a practical tool for use at the point of feeding in a clinic or at home. To facilitate point-of-feeding textural determination, the International Dysphagia Diet Standardization Initiative (IDDSI) framework introduced both standardized descriptions of food textures and readily accessible, low-tech clinical testing measures that allow for the classification of food textures into objective categories.

IDDSI was made widely available in 2015 with the goal of increasing the safety of swallowing for people of varying ages, care settings, and cultures [22]. The developers sought feedback from over 3000 international stakeholders in which a final graphic was created. This final framework consisted of eight continuous objective categories represented by inverted triangles that distinguished “foods” from “drinks” that had distinct colors, numbers, and labels that correspond to standardized characteristics of foods, such as thickness, adhesiveness, and cohesiveness. Levels 5–7 were also labeled as “transitional foods” as their properties potentially cross multiple levels if exposed to saliva or temperature changes [23]. Unlike previous mechanisms to classify food textures and liquid thicknesses that were typically country-specific, this framework is purposefully designed to provide global methods of testing and nomenclature [23]. Furthermore, the IDDSI framework is considered as a “description” of a food/liquid at the point of serving, rather than a mechanism to predetermine a food or drink that is part of a specific patient diet [24]. For example, Stevens and colleagues (2022) attempted to develop a “standardized” recipe for thickening formula with infant cereal without success. An examination of 90 different formulas revealed that cereal and formula have property differences, not IDDSI testing variability, suggesting that one recipe may “not be the ideal practice standard” [25]. This shift from a descriptive to prescriptive mindset is critical in the field as it underscores the importance of examining foods at the time of consumption. Although content and construct validity questions still surround the framework, its use in clinical applications is relevant [26]. Since the inception of the IDDSI framework, a body of literature has emerged that has been relevant to examine a number of clinical questions. For example, the IDDSI framework has been proposed to be an effective means to examine the oral processing of the bolus [27], which was recently examined in healthy adults. Bandini et al. (2022) [28] found that the ready-to-swallow boluses were chewed to at least a soft and bite-sized consistency (level 6). This preliminary work provides clinicians with valuable information regarding food items that may represent choking hazards. The IDDSI framework has also been used to demonstrate that older adults requiring textured modified foods in a long-term care facility were eating foods that were more challenging than what had been prescribed, leading to greater choking risks and decreased overall consumption [29]. Furthermore, this framework has provided a means to consistently describe products such as contrast material for videofluoroscopic examinations [24], liquids thickened with xanthan gum [30], and liquids thickened with pureed foods in a population of pediatric patients with dysphagia [31].

**Table 1:** Parenting website definitions of baby food stages and expected ages for consumption (adapted from [32]).

Baby Food Stage	Expected Age of Use	Parental Expectation
Stage 1	4–6 months	Smooth, runny/thin single-ingredient puree foods, appropriate for beginners
Stage 2	6–9 months	Slightly thicker combination puree foods that might include yogurt, grains, and protein/meats
Stage 3	9+ months	Thick, combination puree foods with soft-cooked pieces of food embedded in the puree that will require some chewing

Given the importance of understanding the hierarchical nature of food textures for the promotion of typical feeding development and safe oral intake for infants and children with dysphagia, it is imperative that commercially available foods marketed for infants and children have accurate and reliable descriptions of the food textures. Current manufacturer “staging” on commercially available food labels provides generally accepted descriptions of the food properties but cannot be guaranteed as accurate against any established hierarchical food framework (such as IDDSI) or reliable among different manufacturers. Therefore, the aims of this project were as follows:

Establish the objective flow or textural characteristics for commercially available baby foods marketed as Stages 1–4 by the manufacturer using the IDDSI clinical testing measures.

Analyze differences in the flow or textural characteristics between “stage”-matched baby foods produced by popular manufacturers in the United States.

Determine if the flow or textural characteristics of commercially available “staged” baby foods are significantly influenced by packaging and delivery methods (jar vs. pouch baby foods).

## 2. Materials and Methods

### 2.1. IDDSI Framework Considerations

**Figure 1:**
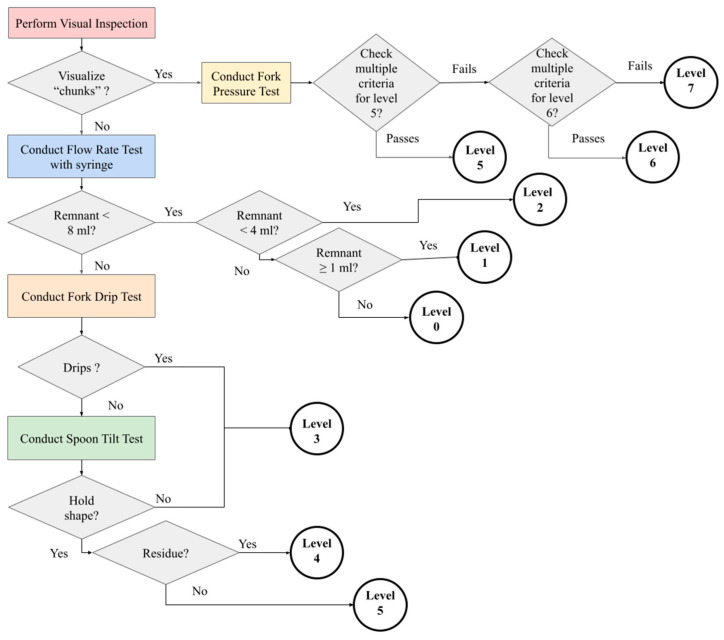
A summary of the IDDSI framework flow as utilized in the study. Upon initial visual inspection of the selected sample, research teams determined what IDDSI test needed to be completed to most accurately categorize the sample into an IDDSI level. These tests included the fork pressure test, the flow rate test, the fork drip test, and the spoon tilt test. Based on the behavior of the sample during the test, either an IDDSI level could be determined or the next IDDSI test would be completed.

At the time of this study, the previously published literature was explored and yielded no information relating the LSs to clinical measures. In an effort to characterize this relationship, the authors conducted this study to examine and evaluate the characteristics of room-temperature baby foods from national brands that were available in two distinct regions of the United States (Ohio and Alabama). This characterization was conducted based on published IDDSI methods [23] available at the time of the study. The study did not incorporate the updated (July 2019) IDDSI Framework. This framework utilizes equipment that is generally available in a clinical setting for universal application. Food was tested utilizing a stopwatch, 10 mL syringes (Becton, Dickinson and Company, Franklin Lakes, NJ, USA with a measured length of 61.5 mm from the from the 0 line to the 10 mL line), metal forks (15 mm across all tines, 4 mm between tines), and metal spoons (length of 2 in, width of 1 1/6 in). Figure 1 depicts a summary of the flow of operations in this framework. A comparison of methodologies utilized by the two research teams is presented in Table 2.

### 2.2. Sampling/Set-Up of Research

For this study, research teams from the University of Alabama Feeding Development and Disorders (FeDD) Laboratory (UA-Tuscaloosa, AL) and Miami University Dysphagia Laboratory (MU-Oxford, OH) conducted parallel, independent experiments to achieve the previously outlined research aims from the fall of 2018 to the spring of 2019. For the MU group, four research assistants (three undergraduate students, one graduate student) were assigned to two research teams (Team A and Team B) to conduct the IDDSI tests on the selected samples. Both Team A and Team B completed 5 trials per product. For the UA group, seven research assistants (six undergraduate, one graduate student) worked as one group and completed 10 trials per product. Team A and Team B from MU and the one group from UA were all blinded to brand, LS, packaging type, and ingredients.

Researchers from UA compiled a comprehensive inventory list of baby foods available in their respective region of the United States from two nationally recognized manufacturers (Gerber, Freemont, MI, USA and Beech-Nut, Amsterdam, NY, USA). Prospective samples included various food classes such as fruits, vegetables, proteins, and blends at manufacturer’s Stages 1, 2, 3, and 4 in various packaging types, including glass jars, plastic containers, and pouches. Resource constraints and the a priori estimates of the sample needed to provide adequate power for statistical analysis resulted in a randomly selected, representative sample of the available baby foods (based on container type, ingredients, and manufacturer’s categorization) [33]. The distribution of the selected samples was representative of products available for purchase at time of sampling. Once the foods were selected by UA, the list was sent to MU. The foods were purchased in each local geographical region of the United States in order to determine if there were regional differences in manufacturer product output. A small amount of the products tested were unavailable for a direct match. Any food that could not be found in Ohio (4 total samples) was substituted with a similar sample from the same food class, packaging, and LS. By design, the MU group added a third exclusively marketed “organic” brand (Earth’s Best, Hain Celestial Group, Hoboken, NJ, USA) to be examined. A comprehensive inventory list of available baby foods within the Earth’s Best brand was compiled, and a representative sample was randomly selected using the same features as Gerber and Beech-Nut.

Prior to testing, the selected samples were de-identified and randomized using a randomization function in Microsoft Excel (Office 2019) by a faculty member or graduate assistant, who were not blinded to the protocol. Both UA and MU testing researchers were blind to brand, LS, packaging types, and ingredients. As per the available IDDSI guidelines [23], the teams utilized standardized equipment to conduct IDDSI testing methods, including a stopwatch, 10 mL syringes (Becton, Dickinson, and Company, with a measured length of 61.5 mm from the from the 0 line to the 10 mL line), metal forks (15 mm across all tines, 4 mm between tines), and metal spoons (length of 2 in, width of 1 1/6 in). All testing sessions were recorded using a video recording device, coded, and saved for reliability purposes.

### 2.3. Baby Food Testing Methods

In order to characterize the foods for comparison, UA and MU laboratories utilized and interpreted research published by IDDSI [23], which was the available method at the time of this research, to create detailed systematic testing protocols. Researchers from each laboratory conducted prescribed IDDSI testing methods systematically to identify final IDDSI levels of selected samples. Samples were tested ten times each by each laboratory. The two laboratories were located in different regions of the United States separated by 750 km. Food samples were acquired locally in each region in order to compare any regional differences in food textures for similarly labeled foods. The foods were purchased in local grocery stores. Grocery retailers vary by region in the United States and have their own supply chains, which may provide similar labelled foods from regional manufacturers. Furthermore, regional consumer preferences influence product availability. Due to regional differences in baby food availability, some foods could only be tested in one region by that regional research team.

### 2.4. Reliability Measures

Video recordings were re-watched for reliability measures. MU determined intra-rater and inter-rater reliability by calculating the percent agreement of IDDSI levels within research at the respective lab teams and between UA and MU research teams. The intra-rater reliability was determined by identifying the number of trials that had the same IDDSI level determination for each sample within a single research team. For example, for a single sample, if Team A (MU) determined a sample to be an IDDSI level 3 (liquidized) for 3 trials and IDDSI level 4 (pureed) for 2 trials, the intra-rater reliability would be 60% (3 of 5 trials were similar). This process was repeated for each research team (2 from MU and 1 from UA) and all samples. Following the determination of intra-rater reliability, the inter-rater reliability was determined. The inter-rater reliability was calculated by identifying the total number of trials that had the same IDDSI level determination for each sample between the two research teams. To calculate this measure, researchers combined the intra-reliability measures from each team. For example, if Research Team A’s intra-rater reliability was 80% for a single sample and Research Team B’s intra-rater reliability was 60% for the same sample, the combined interrater reliability would be 70%. Samples identified to have low intra-rater and/or inter-rater reliability (<80%) were selected to have the videos taken during trials re-watched by independent raters for accuracy.

### 2.5. Statistical Analysis

Descriptive statistics were employed to characterize baby food samples. Frequencies and percentages were used to describe instances of the IDDSI categories. To compare the flow or textural characteristics of baby foods, the means and standard deviations were obtained. Furthermore, the relationships between variables were described by chi-squared tests for independence, ANOVA F-tests, and Kruskal–Wallis tests. Intra- and inter-rater reliability measures were calculated using intraclass correlation coefficient and Fleiss’ Kappa, respectively. All analyses were conducted with R version 4.2.2.

## 3. Results

### 3.1. MU Results

A representative sample of the regionally available baby foods in each of the categories (presentation, ingredients, and manufacturer’s categorization) was collected. A total of 171 baby foods were identified as possible samples. Samples were selected from three national brands (Gerber, Beech-Nnut, and Earth’s Best Organics). Once the samples were identified, the MU researchers then randomly selected approximately 30% of the regionally available samples (N = 66). Intra-class correlation coefficients for each team were 0.95 and 0.94. The Fleiss’ Kappa for inter-team agreement between the two teams was found to be 0.485.

A one-way univariate ANOVA was conducted to compare the effect of (a) brand, Gerber and Beech-Nut; (b) marketed stages; and (c) brand and marketed stages to the IDDSI level of developmental first foods. There was no significant relationship between (a) brand and IDDSI level, F (1, 33) = 3.271, *p* = 0.080; (b) marketed stage and IDDSI level, F (3, 33) = 0.736, *p* = 0.538; or (c) brand and marketed stages to IDDSI level, F (2, 33) = 0.651, *p* = 0.528.

A further descriptive analysis was completed to look at textural and flow characteristics related to packaging type. To make this comparison, bootstrapped 95% confidence intervals were constructed. Foods identified as IDDSI level 4 (pureed) were compared to foods identified as IDDSI level 5 (minced and moist) for textural differences and IDDSI level 3 (liquidized) for flow differences. A 95% confidence interval on the difference in the proportion of baby foods classified as level 4 (pureed) as compared to level 5 (minced and moist) for pouches was between 68.2% less and 10% more than the proportion of baby food in jars/plastic, indicating no difference between the two package types for their textural differences. Similarly, the 95% confidence interval on the difference in the proportion of baby food classified as level 3 (liquidized) as compared to level 4 (pureed) in pouches was between 8.7% less and 16.6% more than the proportion of baby food classified in jars/plastic, indicating no significant difference between pouches and jars/plastic in the proportion of baby food classified as lower flow.

The most prevalent IDDSI level for these samples of data was level 3 (liquidized) with 74.7% of the samples falling under this IDDSI level. Level 4 (pureed) was the second highest seen at 12.7%, level 5 (minced and moist) at 9% and both level 2 (mildly thick) and level 6 (soft and bite-sized) at 1.8%. Breaking down IDDSI levels by LS revealed that Stage 3 was the only LS without IDDSI level 3 (liquidized) being the most prevalent and with IDDSI level 5 (minced and moist) being most common at 43.8%. Stage 2 and Stage 4 had IDDSI level 3 (liquidized) as the most common at 85.8% and 81.8%, respectively. After combining IDDSI levels 2 (mildly thick) and 3 (liquidized), and 5 (minced and moist) and 6 (soft and bite-sized), the chi-squared test for independence revealed a relationship between stage and IDDSI levels (*p* = 0.000). Running the same test between brand and IDDSI level revealed there was not a significant association (*p* = 0.057).

### 3.2. UA Results

A representative sample of the regionally available baby foods in each of the categories (presentation, ingredients, and LS) was collected. A total of 74 baby foods were identified as possible samples from two brands, Beech-Nut and Gerber. An intraclass correlation coefficient was found to be 0.847.

A one-way univariate ANOVA was conducted to compare the effect of (a) brand, (b) LS, and (c) brand and LS to the IDDSI level of developmental first foods. There was no significant relationship between (a) brand and IDDSI level, *F* (1, 33) = 3.271, *p* = 0.080; (b) LS and IDDSI level, *F* (3, 33) = 0.736, *p* = 0.538; or (c) brand and LS to IDDSI level, *F* (2, 33) = 0.651, *p* = 0.528. Additional analysis was conducted using the Kruskal–Wallis test, which determined spoon tilt test scores were not significantly different for measures of flow characteristics (X^2^ (1) = 0.610, *p* = 0.435) or textural characteristics (X^2^ (1) = 0.265, *p* = 0.607) when comparing samples from Beech-Nut and Gerber. Median brand category scores were not statistically different with respect to flow characteristics (X^2^ (3) = 1.640, *p* = 0.650) or textural characteristics (X^2^ (3) = 3.371, *p* = 0.338). Spearman’s rank-order correlation determined that there was no significant correlation between LS and IDDSI level *r_S_* = 0.094, *p* = 0.575. IDDSI levels of samples ranged from level 1 (slightly thick) to level 5 (minced and moist).

A descriptive analysis was also conducted to determine the mean differences in residual product from the flow test between glass jars, plastic containers, and pouches. The plastic containers (*M* = 9.8) and the glass jars (*M* = 9.5) fell within the same IDDSI level and were consequently consolidated into one category and compared to the pouches. A one-way univariate ANOVA was conducted to determine if thickness of developmental first foods was different according to packaging and no statistical difference was found, *F* (1, 38) = 4.047, *p* = 0.051. The Kruskal–Wallis test determined the difference in flow characteristics was not statistically significant in pouches versus jars/plastic containers, X^2^ (1) = 0.726, *p* = 0.394.

### 3.3. Combined Results

**Figure 2:**
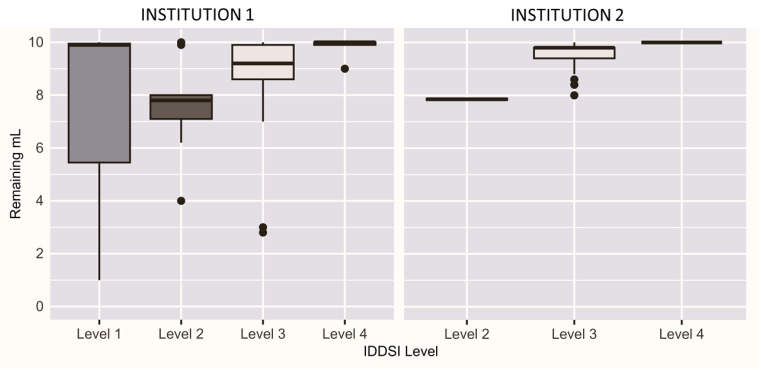
Distribution of the remaining product from the flow test by IDDSI level for both research teams. Two box plots are presented of the remaining product amounts in mL plotted by IDDSI level as obtained by MU and UA during the flow test.

Combining both the MU and UA team results, a total of 69 unique baby foods were examined. Figure 2 presents the distribution of the residual product from the flow test by IDDSI level for both research teams. MU results span four IDDSI levels and UA results span three IDDSI levels.

Table 3 details trial agreement in relation to the brand, LS, and number of ingredient categories for each unique food. Ingredients were categorized into one of five groups: fruit, vegetable, grain, meat, and “other”. Ingredients categorized as ”other” included seasoning, broth, yogurt, cream, or cheeses. Thirty-one (44.9%) baby foods had complete agreement, meaning all trials observed the same IDDSI level. Thirty-two (46.4%) baby foods had two observed IDDSI levels measured. Six (8.7%) baby foods saw three different IDDSI levels observed. An examination of the consistency of IDDSI measurements across trials revealed Earth’s Best brand products had a higher proportion of complete agreement. Stage 1 products, regardless of brand, demonstrated a higher proportion of three different IDDSI levels (30.8%). Four of the five products that spanned three IDDSI levels were Stage 1, three of which contained meat and broth. The exact products that were in complete agreement (Supplemental Table S1), covered two adjacent different IDDSI levels (Supplemental Table S2), or crossed three IDDSI levels (Supplemental Table S3) are included in the Supplemental Materials.

## 4. Discussion

Currently, in the United States, consumers of baby food regularly rely on a subjective numeric labeling (LS) that baby food manufacturers provide on the packaging to make baby food selection decisions. The consumer expects that the LS provides critical information about the consistency of the food that can be directly linked to a specific DS. Additionally, consumers expect congruency of baby food consistencies both within products and across brands. As such, our discussion details three primary findings: (1) variability observed within the same product; (2) variability across products; and (3) clinical implications and related observations to inform our readers for best clinical practice.

### 4.1. Within-Product Variability

When examining the scores of the same product(s), three distinct groups emerged: consistent foods (Group A), small variability foods (Group B), and large variability foods (Group C).

### 4.2. Group A—Consistent Foods

Products were considered “consistent” if all trials across all testing groups determined the same IDDSI level. It is expected that a product would test at the same consistency for all trials with a standard protocol. Table 4 summarizes the IDDSI level distribution of 31 of the 69 products (44.9%) that had complete agreement (refer to Supplemental Table S1). A total of 27 products from all four labelled stages manifested as IDDSI level 3 (27 products). This result shows no correlation between an increase in label stage, leading to an increase in textural thickness. Additional results from Stage 2 and 3 foods were level 4 (3 products) and level 5 (1 product). All four of these products included some type of grain, including oatmeal, barley, and granola. However, the existence of grain as an ingredient was not a sufficient condition, as two level 3 products also contained oat. The manufacturing processing of the product ingredients may play a role in the IDDSI test behavior during the fork drip test or spoon tilt test (refer to Figure 1). Due to this finding, our team examined the products with measured variation to determine possible causes.

### 4.3. Group B—Small Variability Foods

Almost half of the products tested (46.4%; N = 32) resulted in two adjacent measured IDDSI levels. Table 5 summarizes the distribution of the IDDSI level results of these products. This can be attributed to products that have a consistency that is at a threshold for a given IDDSI test (refer to Figure 1). The majority of these products (62.5%, N = 20) scored both levels 3 and 4 (refer to Supplemental Table S2). These can be the result of slight changes in texture that affect the fork drip and the spoon tilt tests (refer to Figure 1). The examination of the videos for these tests with these products verified that the measures were correct. Three of the products tested in the MU lab had an exact split in the number of trials that scored between two IDDSI levels (refer to Supplemental Table S2, Matched Percentage (MP) = 50), which identifies products that span a range of two textures. The IDDSI results can be utilized to identify the range of the product consistency to ensure patient safety. In fact, baby food often demonstrates characteristics of both a liquid and a puree, which heightens the importance of utilizing the complete span of IDDSI tests (flow test, fork drip test, and spoon tilt test). This is evidenced by the variability in results between UA (no fork drip test), which identified as an IDDSI level 4 (puree), and MU (fork drip test), which identified a level 3 (liquidized) (refer to Supplemental Table S2, the six products in purple).

### 4.4. Group C–Large Variability Foods

Six food products crossed three different IDDSI levels, as summarized in Table 6 (refer to Supplemental Table S3). Three of the foods were meat products, two were from a single vegetable (e.g., carrots, green beans), and one was a combination (beef medley vegetables). Furthermore, these of the six products crossed three labelled stages from the manufacturers. The range of ingredients in this group with greater variability displays the difficulty in trying to apply a systematic recommendation just based on ingredients or stages.

Some products in each of the three groups were identified as IDDSI level 5 (minced and moist); chunks were clearly found in some of these products, and these products seemed in line clinically with our “mixed” consistency foods. The authors recommend to clinicians that any baby food observed to have chunks be strained for those infants or patients who cannot developmentally handle higher complexity foods.

**Figure 3:**
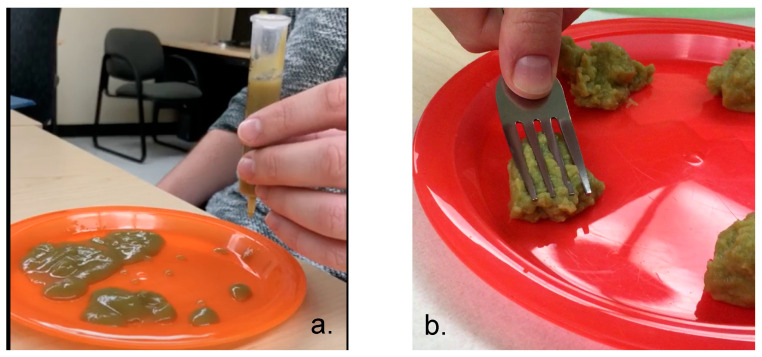
Evidence of the same green bean product from two different containers that were purchased from the same store on the same day revealed (**a**) a more liquid consistency that allowed for the product to drip from the syringe versus (**b**), which contained visible chunks.

One of the single-ingredient foods, green beans, displayed large variability in IDDSI levels. It has been stated that green beans have a unique cell wall structure that makes them particularly difficult to cook and blend [34]. To illustrate, we provide a picture of the same green bean product from two different containers that were purchased from the same store on the same day (Figure 3). It is evident that, in Figure 3a, the product from the container was more of a liquid and, in Figure 3b, clear chunks are observed. Clinicians and caregivers should critically evaluate this product and make safety judgements accordingly.

### 4.5. Across-Product Variability

The findings related to flow and textural characteristics do not support our hypothesis that the LSs are reflective of measured flow characteristics. After completing the study, the authors were unable to pinpoint a single characteristic used by manufacturers to classify baby foods into LSs. In one situation, LS classification correlated with the volume of food in the containers (Gerber Stage 1 and Stage 2 apples) and, in another case, with single-food products (all Stage 1 foods from all brands). From a brand perspective, our data indicate that Earth’s Best (an exclusively organic brand) was significantly different from Gerber and Beech-Nut, suggesting organic brands may have different processing protocols.

Our results show that the brand and LS cannot predict flow and texture characteristics. We found that 75% of the baby foods (regardless of stage) scored a 3 (liquidized) on the IDDSI scale, and four of the six products categorized as large variability foods were labeled as Stage 1. Due to knowledge of maturation of oral–motor skills, one would expect baby food thickness to progress in a linear manner from Stage 1 to Stage 4. This logical progression was not observed in the results. Clinically, these unfulfilled expectations can result in unsafe or inappropriate presentation of foods to children. Furthermore, during the course of this study, Gerber changed their LSs from a numeric classification to gross motor milestones. This revised LS classification system does not consider the needs of many children with dysphagia who may not meet established gross-motor milestones in the same timeframe as their typically developing peers. For example, many children with dysphagia require postural support and other therapeutic aids to participate in daily activities, like feeding. To navigate feeding development for children with dysphagia, the general use of typical gross-motor milestones may be confusing and/or misleading for caregivers.

Another unexpected finding with tremendous clinical relevance involves the content of the food samples used by the manufacturers. It appears that mainstream products do not regularly consider the type of foods that are safe for older infants to consume. Food allergens occur in children and adults world-wide with substantial geographical differences. Highly allergic foods include cow’s milk, eggs, tree nuts, peanuts, shellfish, wheat, soy, and fish [35]. However, other foods in other geographical regions of the world that are also considered include peaches, apricots, strawberries, plums, apples, pears, kiwis, bananas, tomatoes, corn, cauliflower, and sesame [36]. In most Westernized countries, food allergies occur in 6–10% of infants [37]; however, children with feeding disorders experience an increased occurrence of food allergies (40% compared to 8% in children without Pediatric Feeding Disorder) [38]. Current CDC guidelines suggest new foods should be introduced to young children one at a time to rule out potential allergens, and this introduction of potentially allergenic foods should be on a schedule with other new foods [39]. Many of the foods selected for this research included more than one ingredient, sometimes up to five ingredients, including foods known to be highly allergenic globally.

## 5. Strengths and Limitations of the Current Research

The previous evaluation of baby food characteristics utilizing frameworks (i.e., IDDSI) is limited. Thus, the strengths of the study include the systematic selection and evaluation of the selected samples using an established protocol; blinding of all research assistants to brand, ingredient, and stage of the baby foods; testing a variety of available baby foods; and re-watching trial videos for accuracy.

Despite the strengths of this study, both external and internal limitations exist. The primary internal limitation is that, although both the UA and MU teams followed similar protocols, the UA group did not include the fork drip test in their experiments. Both teams followed the website guidance but, because of the inherent nature of baby food (most can be classified in general as liquid (liquidized) or solid (puree)), the teams interpreted the 2017 guidelines differently. However, since 2017, new guidelines have been updated to address this ambiguity.

Additionally, the authors recognize that cohesion and adhesion are not static properties once they are exposed to salivary amylase and lingual lipase. Salivary amylase, which begins the digestion of starches into sugars, reaches adult levels by 3 months of age [7]. Lingual lipase, which participates in lipid digestion and is secreted by the posterior lingual serous glands, is present in preterm and term infants with a gradual decline [7]. Although the impact of the influence of salivary enzymes in the developing infant is beyond the scope of this paper, future research in this area would be beneficial to practicing clinicians. At this time, there is limited research that examines the role, if any, in the salivary digestive enzymes during teething, in infants administered reflux medications or infants who are non-oral during the first 3 months of life.

In the future, samples of baby food should be tested at varying temperatures, chilled and warmed, to capture the behavior across the spectrum of possible temperatures of food presentation. This is particularly important as baby food is not only served at room temperature, but can be chilled or heated as well. Secondly, professionals should collaborate with baby food manufacturers or appropriate governing agencies to develop labeling guidelines that correspond to objective criteria.

## 6. Conclusions

Caregivers and clinicians are responsible for the presentation of safe and appropriate transitional foods during a child’s milk-weaning process. Current “stage”-based baby food labels do not accurately convey the product’s textural characteristics, which can influence the safety and efficiency of oral intake. Based on the results of this study, it is recommended that speech–language pathologists, caregivers, and other health care providers learn and utilize standardized testing protocols on individual foods and liquids offered to children that have dysphagia.

The key messages of this paper are as follows:

Current “stage”-based baby food labels do not accurately convey the product’s textural characteristics, which can influence the safety and efficiency of oral intake.

New foods should be introduced to young children one at a time including foods known to be highly allergenic globally.to rule out potential allergens since some foods in our sample included as many as five ingredients. 

## Figures and Tables

**Table 2:** Methodologic comparisons from the University of Alabama (UA) Feeding Development and Disorders (FeDD) Laboratory versus Miami University (MU) Dysphagia Laboratory.

Similarities	Differences
Sampling, Set Up, and Procedures	
➢ Beech-Nut and Gerber; ➢ Random sampling of regionally available baby foods, including all manufacturing stages (1–4), packaging types (pouches/glass jars/plastic containers), and available ingredients, was conducted; ➢ All samples were de-identified and randomized; ➢ Blinding to sample; ➢ Each sample was tested 10 times each.	➢ MU: Earth’s Best brand in addition to Beech-Nut/Gerber; ➢ UA required training session regarding learning IDDSI protocols with criteria for researcher “passing”; MU required training session, no hard criteria set; ➢ UA: 6 individual testers; MU: 2 research teams (total of 4 testers); ➢ UA testers blinded to purpose.
IDDSI Testing Methods	
➢ Utilized guidelines published by IDDSI to develop testing protocols; ➢ Syringe flow test methods identical; ➢ Spoon tilt test methods identical.	➢ Initial step of the testing process was different (UA started with the flow rate test for every sample-even mixed consistencies; MU inspected for particles first and then used a guiding document to make testing decisions); ➢ MU conducted Fork Drip Test; UA did not; ➢ Fork pressure test varied: MU: thumb blanching method; UA: Iowa Oral Performance Instrument.
Reliability (Distinguish between inter- versus intra-rater reliability)	
➢ Recorded testing of samples; reviewed video for reliability.	➢ Used different processes for reliability (UA: 25% of all recorded samples; MU reviewed video of trials with low reliability) ➢ MU determined intra- and inter-rater reliability; UA only determined intra-rater reliability.

**Table 3:** Trial agreement for the total sample of baby food products tested and subdivided by brand, stage, number of ingredient categories, and summary of ingredient categorization.

	Total Products	Complete Agreement		Split (2) Agreement		Split (3) Agreement	
		N	%	N	%	N	%
Overall	69	31	44.9	32	46.4	6	8.7
Brand							
Beech-Nut	20	6	30	12	60	2	10
Earth’s Best	23	17	73.9	3	13	3	13
Gerber	26	8	30.8	17	65.4	1	3.8
Stage							
1	13	3	23.1	6	46.2	4	30.8
2	43	22	51.2	20	46.5	1	2.3
3	8	4	50	3	37.5	1	12.5
4	5	2	40	3	60	0	0
Number of Ingredient Categories							
1	33	15	45.5	16	48.5	2	6.1
2	28	12	42.9	12	46.4	4	10.7
3	7	4	57.1	3	42.9	0	0
4	1	0	0	1	100	0	0
Summary of Ingredient Categorization							
1	33	15	45.5	16	48.5	2	6.1
2+	26	16	44.4	17	47.2	3	8.3

**Table 4:** Summary of products with complete IDDSI score agreement across every product trial. Stage is the commercially labelled stage. The distribution indicates the number of products that resulted in each of the six IDDSI levels. Each product entry is based on a minimum of ten trials for that product.

Label Stage	Brand	# of Products	IDDSI Level Distribution (# of Products)					
			1	2	3	4	5	6
1	Beech-Nut	1			1			
1	Gerber	2			2			
2	Earth’s Best	15			15			
2	Beech-Nut	3			2	1		
2	Gerber	4			3	1		
3	Earth’s Best	2			1		1	
3	Beech-Nut	2			1	1		
4	Gerber	2			2			

**Table 5:** Summary of products with split agreement between two IDDSI score levels. Stage is the commercially labelled stage. The distribution indicates the number of products that resulted in each of the six IDDSI levels. Each product is represented in two adjacent levels.

Label Stage	Brand	# of Products	IDDSI Level Distribution (# of Products)					
			1	2	3	4	5	6
1	Earth’s Best	1			1	1		
1	Beech-Nut	3		1	2	1	1	1
1	Gerber	2			1	2	1	
2	Earth’s Best	2			2	2		
2	Beech-Nut	7		3	6	4	1	
2	Gerber	11		2	11	9		
3	Beech-Nut	1			1	1		
3	Gerber	2					2	2
4	Beech-Nut	1		1	1			
4	Gerber	2			2	2		

**Table 6:** Summary of products that crossed three different IDDSI levels. Stage is the commercially labelled stage. The distribution indicates the number of products that resulted in each of the six IDDSI levels. Each product is represented in multiple levels.

Label Stage	Brand	# of Products	IDDSI Level Distribution (# of Products)					
			1	2	3	4	5	6
1	Earth’s Best	2	2	2	2			
1	Beech-Nut	2		1	2	1	2	
2	Gerber	1			1	1	1	
3	Earth’s Best	1			1		1	

## Data Availability

The original contributions presented in this study are included in the article/Supplementary Materials. Further inquiries can be directed to the corresponding author.

## Supplementary Materials

The following supporting information can be downloaded at: https://www.mdpi.com/article/10.3390/foods14101771/s1. Table S1: Products with complete IDDSI score agreement across every trial; Table S2: Products with split agreement between two IDDSI score levels; Table S3: Products that cross three different IDDSI levels.
