# Supplementary material for: Textural Flow Analysis of United States Commercially Available Baby Foods: Packaging and Delivery Method Comparisons by the International Dysphagia Diet Standardization Initiative Framework
Source: Foods. 2025 May 16;14(10):1771. doi: 10.3390/foods14101771 (PMC12110830; doi:10.3390/foods14101771)
Supplement: Supplementary file 1 [file foods-14-01771-s001.zip › foods-3571107-supplementary.pdf]

Table S1: Products with complete IDDSI score agreement across every trial. Stage is the commercially labelled stage. Median IDDSI levels are based on the recorded range from 1-6. The distribution of the 10 or 20 trials for each product is provided across six IDDSI levels.

					Median IDDSI levels						Distribution of IDDSI level trials					
**Product ID**	**Brand**	**Stage**	**Package**	**Ingredients**	**Com-bined**	**MU Team A**	**MU Team B**	**UA Team**	**Total # of Trials**	**% Matched***	**1**	**2**	**3**	**4**	**5**	**6**
5	Earth's Best	2	glass	apples	3	3	3	NA	10	100	0	0	10	0	0	0
7	Beech-Nut	2	pouch	banana blueberry avocado	3	3	3	3	20	100	0	0	20	0	0	0
11	Gerber	1	plastic	peach	3	3	3	3	20	100	0	0	20	0	0	0
14	Earth's Best	2	glass	apples blueberry	3	3	3	NA	10	100	0	0	10	0	0	0
19	Earth's Best	2	pouch	sweet potato beets	3	3	3	NA	10	100	0	0	10	0	0	0
20	Earth's Best	2	pouch	blueberry banana flax oat	3	3	3	NA	10	100	0	0	10	0	0	0

21	Earth's Best	2	glass	carrots	3	3	3	NA	10	100	0	0	10	0	0	0
22	Gerber	2	plastic	apples	3	3	3	3	20	100	0	0	20	0	0	0
26	Earth's Best	2	glass	sweet potato chicken	3	3	3	NA	10	100	0	0	10	0	0	0
28	Earth's Best	2	pouch	sweet potato apple	3	3	3	NA	10	100	0	0	10	0	0	0
29	Earth's Best	2	pouch	sweet potato cinnamon flax oat	3	3	3	NA	10	100	0	0	10	0	0	0
30	Beech-Nut	2	glass	apple	3	3	3	3	20	100	0	0	20	0	0	0
31	Gerber	2	plastic	pea carrot spinach	3	3	3	3	20	100	0	0	20	0	0	0
36	Gerber	2	plastic	vegetable chicken	3	3	3	3	20	100	0	0	20	0	0	0
42	Gerber	2	pouch	banana blueberry blackberry oatmeal	4	4	4	4	20	100	0	0	0	20	0	0
44	Earth's Best	3	glass	apple cinnamon oatmeal	5	5	5	NA	10	100	0	0	0	0	10	0

45	Earth's Best	2	pouch	apple strawberry	3	3	3	NA	10	100	0	0	10	0	0	0
50	Earth's Best	2	glass	pears mango	3	3	3	NA	10	100	0	0	10	0	0	0
51	Earth's Best	3	pouch	chicken casserole vegetables rice	3	3	3	NA	10	100	0	0	10	0	0	0
52	Earth's Best	2	glass	banana mango	3	3	3	NA	10	100	0	0	10	0	0	0
57	Beech-Nut	3	glass	sweet potato barley	4	4	4	4	20	100	0	0	0	20	0	0
59	Earth's Best	2	glass	peach oatmeal banana	3	3	3	NA	10	100	0	0	10	0	0	0
60	Earth's Best	2	pouch	banana raspberry brown rice	3	3	3	NA	10	100	0	0	10	0	0	0
61	Earth's Best	2	glass	chicken brown rice	3	3	3	NA	10	100	0	0	10	0	0	0
62	Gerber	4	pouch	peaches cream	3	3	3	3	20	100	0	0	20	0	0	0
64	Earth's Best	2	glass	prunes	3	3	3	NA	10	100	0	0	10	0	0	0

65	Gerber	1	plastic	carrots	3	3	3	3	20	100	0	0	20	0	0	0
101	Beech-Nut	1	glass	turkey & broth	3	NA	NA	3	10	100	0	0	10	0	0	0
102	Beech-Nut	2	glass	banana cinnamon granola	4	NA	NA	4	10	100	0	0	0	10	0	0
104	Beech-Nut	3	glass	just sweet potatoes squash & peas	3	NA	NA	3	10	100	0	0	10	0	0	0
106	Gerber	4	pouch	peach apricot carrot w/ yogurt	3	NA	NA	3	10	100	0	0	10	0	0	0

* percentage of the IDDSI scores that are matched

Table S2: Products with split agreement between two IDDSI score levels. Stage is the commercially labelled stage. Median IDDSI levels are based on the recorded range from 1-6. The distribution of the 10 or 20 trials for each product is provided across six IDDSI levels.

					**Median IDDSI levels**						**Distribution of IDDSI level trials**					
**Product ID**	**Brand**	**Stage**	**Package**	**Ingredients**	**Com-bined**	**MU Team A**	**MU Team B**	**UA Team**	**Total # of Trials**	**% Matched***	**1**	**2**	**3**	**4**	**5**	**6**
1	Beech-Nut	2	glass	peaches	3	3	3	3	19	89.5	0	2	17	0	0	0
2	Gerber	1	plastic	apples	3	4	3	3	20	85	0	0	17	3	0	0
3	Gerber	2	pouch	pear peach strawberry	3.5	3	3	4	20	50	0	0	10	10	0	0
4	Beech-Nut	2	pouch	apple sweet potato pineapple oat	4	3	4	4	20	60	0	0	8	12	0	0
6	Gerber	4	pouch	banana blueberry	4	4	3	4	20	65	0	0	7	13	0	0
8	Beech-Nut	2	glass	green beans	3	3	3	3	20	95	0	1	19	0	0	0
9	Beech-Nut	1	glass	apples	3	4	3	3	20	65	0	0	13	7	0	0

10	Beech-Nut	2	glass	sweet carrots	3	3	3	3	20	95	0	1	19	0	0	0
13	Gerber	2	plastic	peaches	3.5	3	3	4	20	50	0	0	10	10	0	0
15	Gerber	1	plastic	green beans	4	4	5	4	20	75	0	0	0	15	5	0
16	Gerber	2	plastic	carrots	3	3	3	3	20	90	0	0	18	2	0	0
17	Gerber	2	plastic	apple avocado	4	4	3	4	20	65	0	0	7	13	0	0
23	Beech-Nut	1	glass	Beef w/ beef broth	5	5	6	NA	10	60	0	0	0	0	6	4
32	Beech-Nut	1	glass	sweet potatoes	3	3	3	3	20	95	0	1	19	0	0	0
33	Gerber	2	pouch	apple blueberry spinach	3	3	3	4	20	70	0	0	14	6	0	0
34	Beech =Nut	2	glass	apple pumpkin granola	3	3	4	NA	10	60	0	0	6	4	0	0
35	Gerber	2	plastic	macaroni cheese	3	3	3	4	20	55	0	0	11	9	0	0
37	Gerber	2	plastic	carrot mango pineapple	3	3	2	3	20	75	0	5	15	0	0	0

38	Gerber	2	plastic	pear zucchini corn	3	3	3	3	20	90	0	2	18	0	0	0
39	Gerber	2	plastic	apricot mixed fruit	3	3	3	4	20	55	0	0	11	9	0	0
41	Gerber	4	pouch	banana pear zucchini	3	3	3	3	20	85	0	0	17	3	0	0
43	Gerber	2	plastic	pear cinnamon oatmeal	3	4	3	3	20	70	0	0	14	6	0	0
46	Earth’s Best	2	pouch	orange banana	3	3	3	NA	10	90	0	0	9	1	0	0
47	Gerber	3	plastic	banana apple strawberry	5	5	5	5	20	95	0	0	0	0	19	1
48	Earth’s Best	1	glass	peas	3.5	4	3	NA	10	50	0	0	5	5	0	0
49	Gerber	3	plastic	roasted vegetable chicken	6	6	6	7	14	57.1	0	0	0	0	6	8
53	Earth’s Best	3	pouch	beef medley vegetables	4	3	5	NA	10	50	0	0	5	0	5	0
54	Gerber	2	pouch	pear squash	3.5	3	3	4	20	50	0	0	10	10	0	0

56	Beech-Nut	2	glass	apple kale	4.5	4	5	NA	10	50	0	0	0	5	5	0
58	Beech-Nut	4	pouch	yogurt apple pumpkin cinnamon quinoa	3	3	3	NA	10	80	0	2	8	0	0	0
63	Earth's Best	2	pouch	spinach lentil brown rice	3	3	3	NA	10	80	0	0	8	2	0	0
103	Beech-Nut	2	glass	pear kale cucumber	4	NA	NA	4	10	80	0	0	2	8	0	0
105	Beech-Nut	3	glass	just apple & zucchini	4	NA	NA	4	10	90	0	0	1	9	0	0

* percentage of the IDDSI scores that are matched

Table S3: Products that cross three different IDDSI levels. Stage is the commercially labelled stage. Median IDDSI levels are based on the recorded range from 1-6. The distribution of the 10 or 20 trials for each product is provided across six IDDSI levels.

					**Median IDDSI levels**						**Distribution of IDDSI level trials**					
**Product ID**	**Brand**	**Stage**	**Package**	**Ingredients**	**Com-bined**	**MU Team A**	**MU Team B**	**UA Team**	**Total # of Trials**	**% Matc hed***	**1**	**2**	**3**	**4**	**5**	**6**
12	Gerber	2	plastic	greenbean	4	3	5	4	20	50	0	0	5	10	5	0
18	Beech-Nut	1	glass	chicken chickenbroth	4.5	5	5	3	20	50	0	0	8	2	10	0
24	Earth's Best	1	glass	turkey turkeybroth	3	3	3	NA	10	80	1	1	8	0	0	0
25	Earth's Best	1	glass	chicken chickenbroth	2	2	2	NA	10	60	2	6	2	0	0	0
27	Beech-Nut	1	glass	carrots	4	5	5	3	20	50	0	1	9	0	10	0
53	Earth's Best	3	pouch	beef medley vegetables	4	3	5	NA	10	50	0	0	5	0	5	0

*% of mode of the IDDSI score
